# Identifying the Influences on Network Formation in Structural Isomers of Multifunctional Epoxies Using Near-Infrared Spectroscopy

**DOI:** 10.1021/acs.macromol.4c00274

**Published:** 2024-03-29

**Authors:** Matthew B. Whittaker, Joel P. Foreman

**Affiliations:** Department of Materials Science and Engineering, Sir Robert Hadfield Building, University of Sheffield, Sheffield S1 3JD, United Kingdom

## Abstract

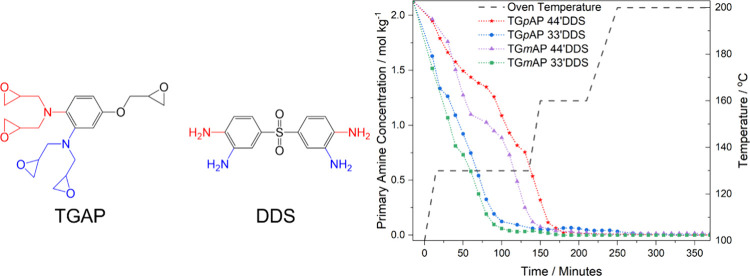

The network formation of four epoxy-rich formulations of the structural isomers of triglycidyl aminophenol and diaminodiphenyl sulfone has been monitored by using two complementary techniques, near-infrared spectroscopy and resin temperature monitoring. The differences between these networks have been described using the concentration of epoxide, primary amine, secondary amine, and tertiary amine functional groups and the actual temperature of the resin compared to the oven temperature during the cure schedule. It was found that initially, the 3,3′-diaminodiphenyl sulfone (33′DDS) formulations were more reactive and primary amines were completely consumed before the 4,4′-diaminodiphenyl sulfone (44′DDS) formulations. Secondary amines were formed more quickly in 33′DDS formulations compared to 44′DDS. The triglycidyl-*meta*-aminophenol (TG*m*AP) formulations consumed secondary amines and produced tertiary amines faster than the triglycidyl-*para*-aminophenol (TG*p*AP) formulations, indicating higher levels of cross-linking occurred earlier in the curing reaction. Etherification occurred much earlier in the TG*p*AP formulations than in the TG*m*AP formulations. Results suggest that internal cyclization occurs in the three meta isomer-containing formulations, and a corresponding lack of this effect in TG*p*AP/44′DDS results in a more homogeneous cross-linked network.

## 2 Introduction

As the demand for composite materials increases, shown by the estimated 2024 annual growth rate at an estimated 3.3%,^1^ so does the need for appropriate matrix materials. Multifunctional epoxy resins cured with diamine hardeners fit this criterion. Their high stiffness, high strength, low shrinkage, good substrate adhesion, and chemical and solvent resistance make them excellent candidates for use as matrices in high-performance composites.^2^ There are numerous different epoxy resins currently in use, such as diglycidyl ether of bisphenol A (DGEBA), tetraglycidyl-4,4′-diaminodiphenylmethane (TGDDM), and triglycidyl aminophenol (TGAP) all having different chemical structures resulting in different mechanical and thermomechanical properties.^3^ These properties determine their suitability for use in composite applications, and many studies have determined and monitored their evolution.

TGAP cured with a diamine hardener such as diaminodiphenyl sulfone (DDS) results in a highly cross-linked 3D network. Cross-linking occurs due to the DDS amine hydrogen functionality (two per amine) and TGAP’s epoxy functionality (three). The two phenylene rings in the amine and the single phenylene ring in TGAP contribute to a stiff backbone, which, when combined with the cross-linking, results in a brittle material. These resins can be toughened using an additional thermoplastic component such as polyethersulfone,^3−5^ phenolic terminated polysulfone,^6,7^ or poly(ether imide).^5^ The additives plasticize the network, resulting in higher fracture toughness while often decreasing stiffness and strength. A different method of toughening may be more suitable, such as altering the network structure by using different structural isomers of resin and hardener. By changing the position of the reactive groups, the reactivity of these groups changes as well as the geometrical position, thus affecting how the network structure can form.^8−10^

**Figure 1:**
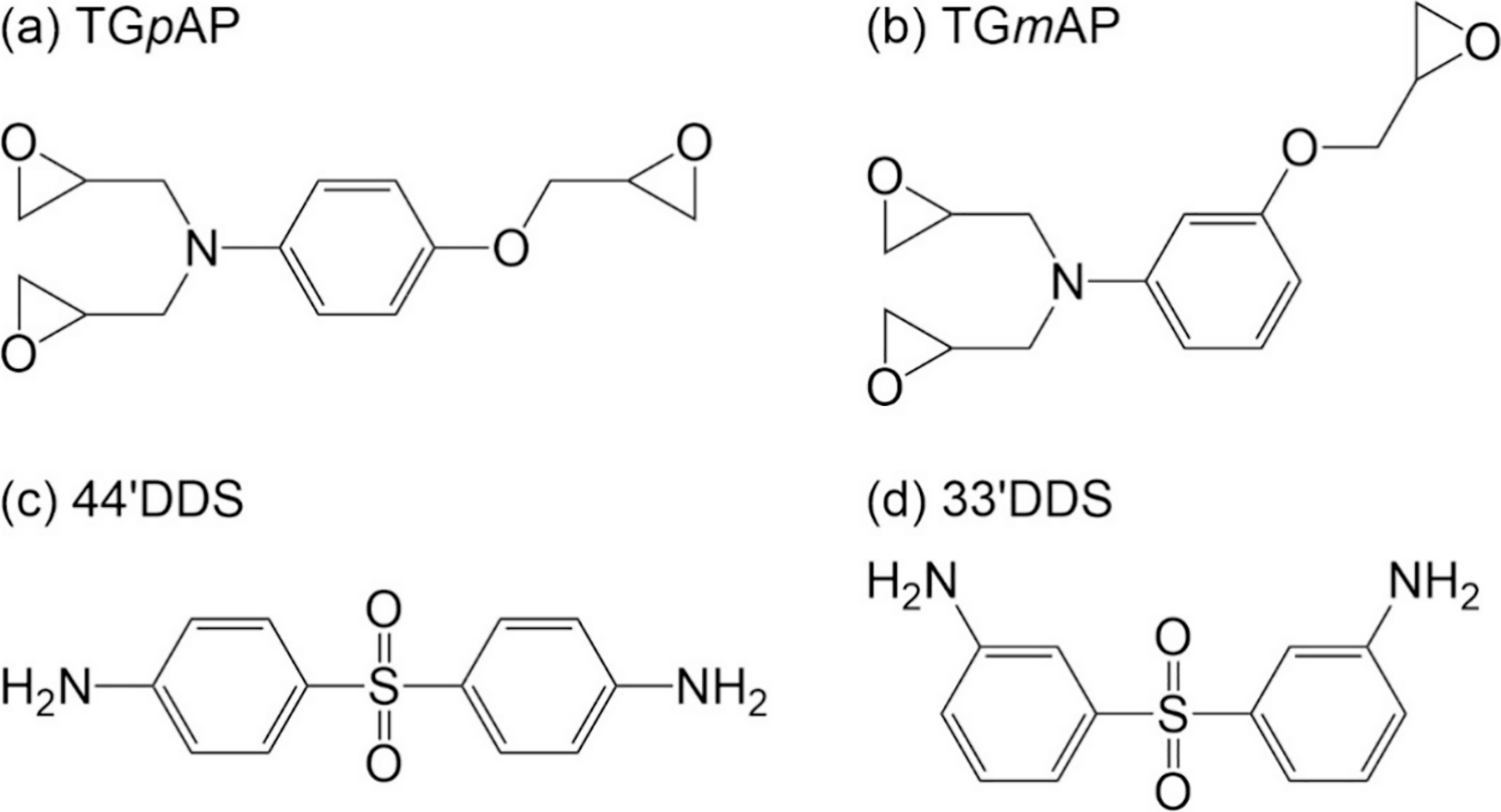
Chemical structures of (a) triglycidyl-*para*-aminophenol (TG*p*AP), (b) triglycidyl-*meta*-aminophenol (TG*m*AP), (c) 4,4′-diaminodiphenyl sulfone (4,4′DDS), and (d) 3,3′-diaminodiphenyl sulfone (3,3′DDS).

Ramsdale-Capper & Foreman looked at how using different structural isomers of TGAP and DDS affects the network structure.^11^ Replacing *para* substituted phenylene rings with *meta* substituted phenylene rings (as shown in Figure 1) resulted in internal antiplasticization. The structural isomers of DDS have also been cured with diglycidyl ether of bisphenol-F (DGEBF), DGEBA, TGDDM and TG*p*AP and their structural properties investigated.^12,13^ Additionally, Frank et al. investigated TG*m*AP cured with 33’DDS^14^ but there have been relatively few studies comparing the two isomers of TGAP with the two isomers of DDS.^15^

Mid-infrared spectroscopy (400–4000 cm^–1^) is a technique that can identify functional groups using fundamental vibrational transitions. It is suitable for general characterization of epoxy resins, but it is difficult to isolate specific functional group bands due to spectral overlap. Near-infrared spectroscopy (12,500–4000 cm^–1^) (NIR) utilizes the nonfundamental vibrational transitions—the overtones (Δυ ≠ 1) and combinations (more than one vibrational transition). This results in a spectrum where the hydrogen-containing bands can be isolated as they have a relatively high intensity compared to non-hydrogen-containing groups and can be used for calculating functional group concentration.^16,17^ NIR is a widely used technique for monitoring the cure of an epoxy resin^7,16−26^ and has been used to determine the concentration of hydrogen containing functional groups (epoxide, primary amine, and secondary amine) during the curing process. From that, it has been possible to identify the specific reactions occurring during network formation in many epoxy systems.

NIR has been used to investigate the effect of isomerism such as Knox et al. investigating network structures of the three regioisomers of DGEBF.^26^ Jackson et al. identified the network effects of using different structural isomers of DDS in DGEBF, DGEBA, and TGDDM resins.^24^ Similarly, Frank and Wiggins used NIR to compare the two isomers of DDS in DGEBF and DGEBA resins and the effect of excess epoxy formulations.^25^ Despite its wide use, we are not aware of NIR having been used to investigate network formation in the structural isomers of both TGAP and DDS together.

**Figure 2:**
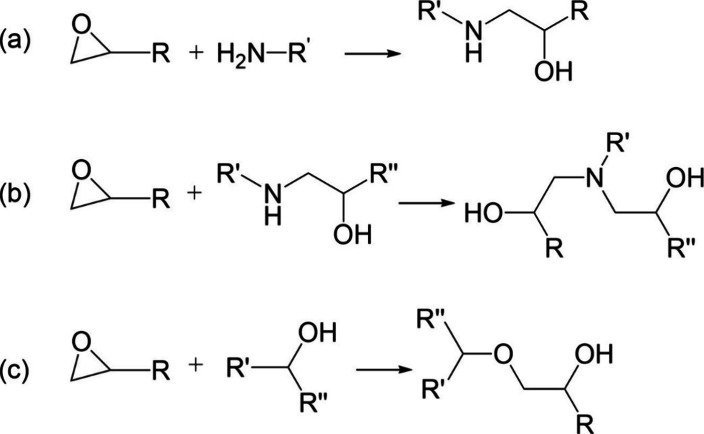
Chemical reactions which occur during an epoxy amine curing process: (a) primary amine, (b) secondary amine, and (c) etherification.

The chemical reactions that occur during the epoxy amine curing process are shown in Figure 2. First, the epoxide ring will react with a primary amine to give a secondary amine and a hydroxyl group. This secondary amine will then react with another epoxide ring to give a tertiary amine and another hydroxyl group. Another reaction that may occur given the correct conditions is etherification, a reaction between a hydroxyl group and an epoxide ring.

Previous studies that have monitored the network formation in epoxy resins using NIR have used “isothermal” cures or an isotherm and a post cure. The chemical reactions shown in Figure 2 are known to have different activation energies, and by using an isotherm, the ability to distinguish between the reactions is therefore limited. In contrast, a more traditional cure cycle with multiple ramps and dwells provides more opportunity to differentiate between the chemical reactions occurring at any given stage in the process. Despite this, multistep cure cycles can allow for increased error due to the temperature dependence of molar absorptivity, which in turn may reduce the accuracy of the NIR analysis.^27^

Another technique that can give insight into the network formation is the actual resin temperature (as opposed to the indicated oven temperature). Bond formation during an epoxy amine reaction is exothermic, such that when the rate of reaction is high, substantial energy is released in the form of heat. This is seen as a higher resin temperature than the oven temperature. We are not aware of any study that has directly monitored the temperature of the resin during cure, but many studies have undertaken differential scanning calorimetry^8,11,28−34^ to identify the thermal events occurring during epoxy cure. By measuring the actual resin temperature, the process is simplified and can more easily be applied to composite curing processes.

The network formation of four different epoxy-rich formulations of TGAP and DDS will be monitored by using near-infrared spectroscopy and resin temperature monitoring. The formation of the networks will then be analyzed and compared to determine the effect that network formation has on the cured resin properties. By understanding how the different structural isomers affect the network formation, fine-tuning of mechanical and thermomechanical properties using resin properties rather than additives can then be achieved.

## 3 Methodology

### 3.1 Materials

The epoxies triglycidyl-*para*-aminophenol (TG*p*AP) and triglycidyl-*meta*-aminophenol (TG*m*AP) were supplied by Huntsman Advanced Materials (as Araldite MY0510 and Araldite MY0610, respectively). The two amines 4,4′-diaminodiphenyl sulfone (44′DDS) and 3,3′-diaminodiphenyl sulfone (33′DDS) were supplied by Thermo Scientific and Huntsman Advanced Materials (as Aradur 9719-1 NL), respectively. Four cured resin formulations were produced and are referred to as TG*p*AP/44′DDS, TG*p*AP/33′DDS, TG*m*AP/44′DDS and TG*m*AP/33′DDS throughout this paper.

### 3.2 Preparation of Epoxy Resins

An epoxy:amine mass ratio of 100:36 was used, an epoxy-rich mixture compared to the stoichiometric ratio of 100:67, which accounts for two issues. First is the significant difference between theoretical and real epoxy group functionality when this is more than two. Second is to ensure that the vast majority of amine groups are reacted, which restricts moisture absorption in the cured resin and is common industrial practice.^11,18^ The resin was heated to 60 °C, the amine was added, and the temperature was then increased to 120 °C and mechanically stirred until the amine dissolved (approximately 10 min). The mixture was degassed in a vacuum oven at 100 °C. For resin temperature profiling, the samples were cast into a 100 mm × 100 mm glass dish to an approximate depth of 4 mm to ensure the resin remained in place and covered the thermocouple and then cured in an air convection oven. Near-infrared spectroscopy required resin to be placed between two glass slides with an ∼0.4 mm PTFE spacer and then cured on a heating stage.

The cure cycle used was as follows: 100 to 130 °C at 2 °C min^–1^ (15 min), 130 °C for 2 h, 130 to 160 °C at 2 °C min^–1^ (15 min), 160 °C for 1 h, 160 to 200 °C at 1 °C min^–1^ (40 min), and 200 °C for 2 h: 6 h and 10 min total curing time.

### 3.3 Resin Temperature Profile

A Pico Technology TC-08 USB thermocouple data logger with an exposed junction K-type thermocouple was embedded in the liquid resin to monitor the actual temperature of the resin, with a separate thermocouple to monitor the oven temperature.

### 3.4 Near-Infrared Spectroscopy

NIR was performed using an Ocean Optics NIRQuest 2500. Transmission mode was used in a range of 11,000 to 4000 cm^–1^ using an integration time of 16 ms and 16 scans to average and 6 cm^–1^ resolution. Samples were prepared using a ∼0.4 mm PTFE spacer attached to a glass slide with high-temperature tape where resin was placed and another slide was set on top, ensuring that the path length remains constant. The sample was then heated by using a Linkam THMS600 heating stage. The NIR spectra were smoothed in OriginPro^35^ and exported to Fityk,^36^ where the bands were deconvoluted and then analysis graphs were generated using OriginPro.

## 4 Results and Discussion

### 4.1 Near-Infrared Cure Monitoring

Varley et al. reported a successful method for using NIR to monitor TGAP cured with DDS, which will be closely followed here.^18^ Beer’s law can be utilized to calculate the concentration of a given functional group.^37^ The path length must be known, and the molar absorption coefficient must be calculated to obtain functional group concentrations. The absorbance value used is the area under the specific functional group peak rather than the absolute absorbance value. An internal standard band is used, where the concentration remained constant throughout the curing reaction. The peak at 5960 cm^–1^ caused by the C–H vibration in the aromatic rings present in TGAP and DDS was used in this study as it is not involved in any curing reactions.

Some functional group bands overlap. To account for this, it is necessary to assume a superposition principle, as shown in eq 1.
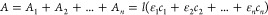
1where *A* is the total absorbance for the band, ε is the molar absorbance coefficient (kg mol^–1^ cm^–1^), *c* is the concentration (mol kg^–1^), *l* is the path length (cm) and *A*_1_*, A*_2_*, A*_*n*_,ε_1_, ε_2_, ε_n_, *c*_1_, *c*_2_, and *c*_*n*_ are component values.

**Figure 3:**
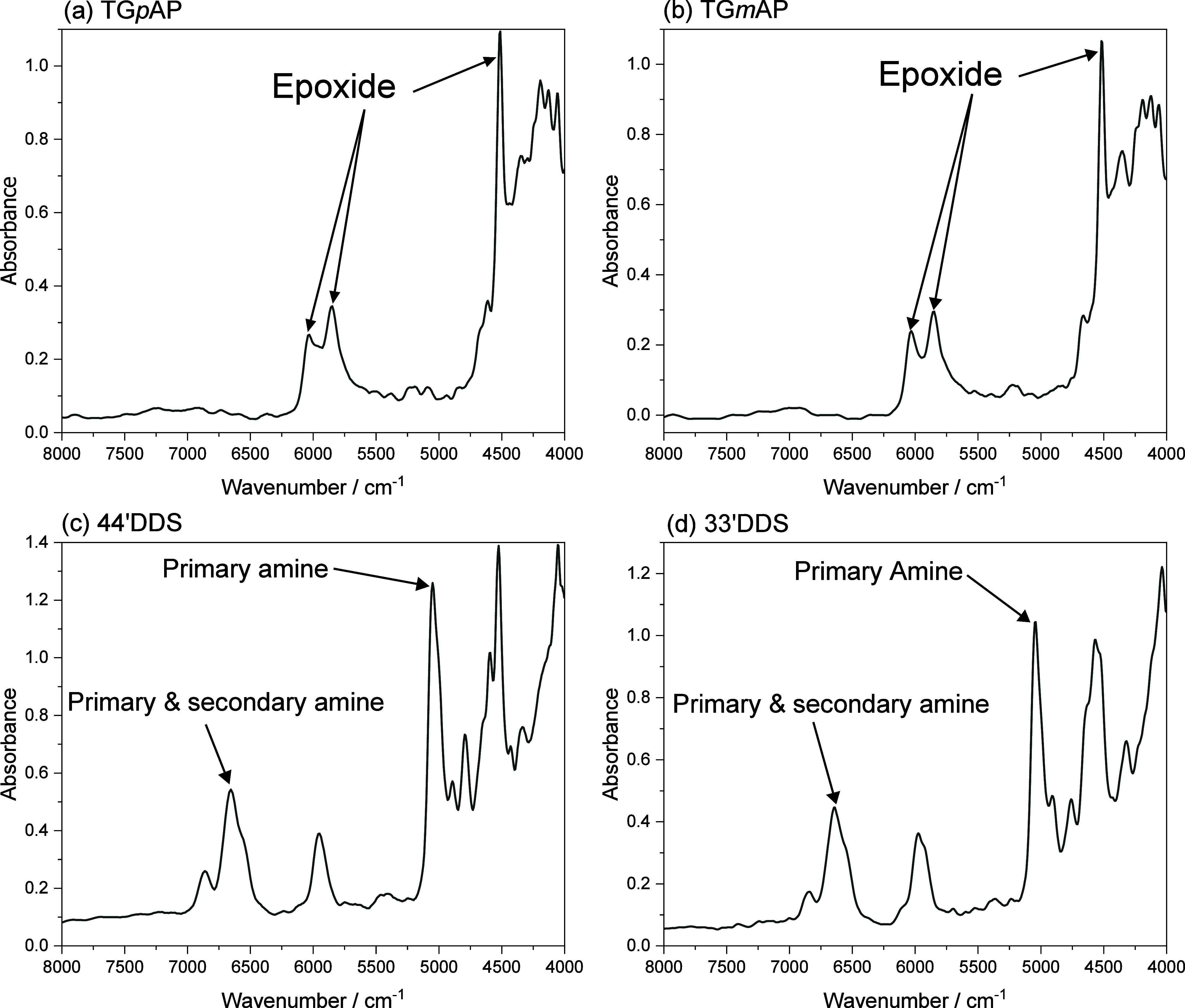
Near-infrared spectra of initial reagents: (a) TG*p*AP (100 °C), (b) TG*m*AP (100 °C), (c) 44’DDS (200 °C), and (d) 33’DDS (200 °C) (smoothed).

**Figure 4:**
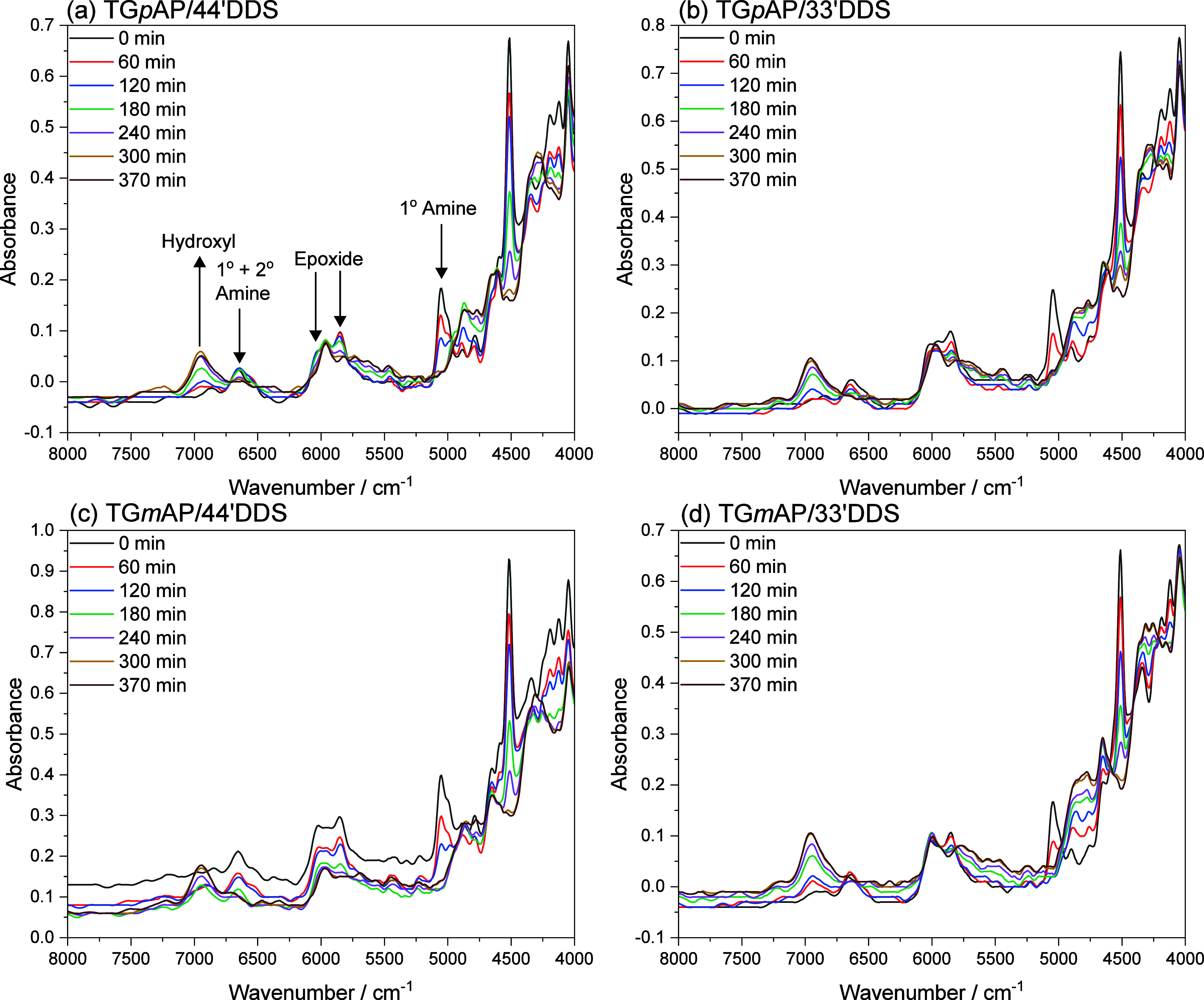
Near-infrared spectra of the curing reaction (100 to 200 °C temperature range) of the four TGAP/DDS formulations (a) TG*p*AP/44′DDS, (b) TG*p*AP/33′DDS, (c) TG*m*AP/44′DDS, and (d) TG*m*AP/33′DDS (smoothed).

Functional group bands were identified using values from literature,^7,19,22^ in particular the bands identified from Varley et al.’s study on TG*p*AP cured with 44′DDS.^18^ Like Varley et al., the bands were first identified using the NIR spectra of the initial reagents, the isomers of TGAP and DDS. The corresponding functional group bands were found in similar positions for each isomer, often at the same wavenumber or differing by the equipment resolution. The NIR spectra shown in Figure 3 present well-isolated bands for some functional groups, but upon mixing, significant overlap occurs, as shown in Figure 4. Poisson et al. used the epoxide CH_2_ peak at 4506 cm^–1^, which had no primary amine overlap in their system,^38^ unlike Min and researchers, who had primary amine overlap at 4535 cm^–1^.^7^ This overlap is common in DDS systems, as was the case here; both 44′DDS and 33′DDS formulations experience epoxide and primary amine overlap. The band assignments used to analyze the isomers of TGAP/DDS can be seen in Table 1. In-depth band assignments have previously been performed and will not be discussed further here.^7,18,22^

**Table 1:** Band Assignments for the Functional Groups of the TGAP and DDS Isomers

**functional group**	**wavenumber/cm**^**–1**^
epoxide C–H	6038 (TG*p*AP) and 6034 (TG*m*AP), 5850 (TG*p*AP and TG*m*AP), and 4517 (TG*p*AP and TG*m*AP)
NH_2_ (1° only)	5059 (44′DDS) and 5047 (33′DDS)
NH (1 and 2°)	6657 (44′DDS) and 6642 (33′DDS)
aromatic C–H	5962 (TG*p*AP and TG*m*AP), 5970 (44′DDS and 33′DDS), 4616 (TG*p*AP), 4683 (TG*p*AP), 4602 (TG*m*AP), 4675 (TG*m*AP)
hydroxyl O–H	∼7000

Figure 4 shows the evolution of the reactive functional group bands during the curing reaction. As the curing process progresses, the area of the specific functional group band changes based on the reactions shown in Figure 2. Some bands required deconvolution, especially the overlapping peaks at 6000 cm^–1^, where at least three bands (an epoxide CH at 5850 cm^–1^, aromatic CH at 5960 cm^–1^, and epoxide CH_2_ at 6038 cm^–1^) overlap. The epoxide CH band at 4517 cm^–1^ is a peak that has been used in previous studies,^7,38^ but due to overlap in that region, it is difficult to deconvolute reliably. Instead, deconvoluting the 6000 cm^–1^ overlapping peaks was a more suitable method for obtaining the epoxide and aromatic band area. This follows the St John and George^19^ and Varley et al.^18^ methods and has successfully produced reliable data, as discussed later.

### 4.2 Molar Absorption Coefficient

Utilizing Beer’s law, molar absorption coefficients, ε, were calculated for the epoxide band at 6038 cm^–1^ and the primary amine bands at 5050 and 6650 cm^–1^. To do this, the initial concentrations of each functional group were calculated using the ratio of TGAP to DDS (100:36) and an epoxy equivalent weight of TGAP (100 g mol^–1^). As the four formulations differ only by functional group position on the ring rather than chemical composition, the epoxide and amine concentrations were the same for each formulation. The initial epoxide concentration was therefore 7.35 mol kg^–1^, and the initial primary amine concentration was 2.14 mol kg^–1^.

The area of the absorbance band was obtained for each different reagent from each NIR spectrum. In its pure form, DDS is a crystalline powder; therefore, to obtain a transmission NIR spectrum, it had to be melted (170 °C). The molar absorption coefficient is temperature dependent, and Varley et al. accounted for this by calculating the coefficient at different temperatures.^18^ In contrast, this study has taken a similar approach to Janisse,^27^ where a scaling factor accounts for the temperature dependence and changes in sample viscosity and refractive index. In this work, it is seen that the internal standard peak changes with temperature throughout the reaction up to 30% over a 100 °C range, a value similar to that of Janisse.^27^ Using the measured path lengths, the resultant molar absorption coefficients can be calculated and are shown in Table 2 where there is a difference between the values for 44′DDS and 33′DDS. The 44′DDS values are similar (assuming the same density values) to those reported by Jackson et al.^24^ While 44′DDS and 33′DDS have the same chemical composition, they have different functional group positioning on the phenyl ring. This allows different resonance structures in the DDS isomers, which interact differently with light, resulting in different molar absorption coefficient values.

**Table 2:** Molar Absorption Coefficients of the Functional Groups of the Isomers of TGAP and DDS

functional group and wavenumber	molar absorption coefficient/kg mol^–1^ cm^–1^
TG*p*AP epoxide CH_2_ (6038 cm^–1^)	33.0
TG*m*AP epoxide CH_2_ (6034 cm^–1^)	38.6
44′DDS primary amine NH (5059 cm^–1^)	125.9
33′DDS primary amine NH (5059 cm^–1^)	147.5
44′DDS primary amine NH (6657 cm^–1^)	81.5
33′DDS primary amine NH (6642 cm^–1^)	82.3
TG*p*AP/44′DDS secondary amine NH (6657 cm^–1^)	140.0
TG*p*AP/33′DDS secondary amine NH (6642 cm^–1^)	157.0
TGmAP/44′DDS secondary amine NH (6657 cm^–1^)	140.0
TGmAP/33′DDS secondary amine NH (6642 cm^–1^)	127.0

Calculating the molar absorption coefficient of the secondary amine band required a more involved procedure. The peak at 6650 cm^–1^ was used as it has been proven to be caused by both primary amine (PA) and secondary amine (SA).^22^ It had to be assumed that there was no tertiary amine formation in the early parts of the curing reactions, which may not be entirely accurate, but without this assumption, there would be no way to calculate the concentration of the secondary amine. Using the superposition principle in eq 1, the sum of the band is given in eq 2.
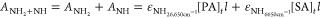
2

Assuming that the tertiary amine concentration is zero in the initial stages of cure, the secondary amine concentration at a given time [SA]_t_ is given by eq 3.
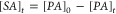
3where [PA]_0_ is the initial primary amine concentration and [PA]_t_ is the primary amine concentration at a given time. Using eqs 2 and 3 and the absorbance value for the band at 6650 cm^–1^, eq 4 can be used to calculate the molar absorption coefficient of the secondary amine band at 6650 cm^–1^.
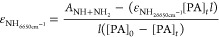
4

Due to the assumptions involved, the secondary amine molar absorption coefficients for each formulation are slightly different and are also reported in Table 2.

### 4.3 Functional Group Concentration Calculations

Epoxide and primary amine concentrations are calculated by using eq 5.
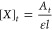
5where [*X*]_*t*_ is the functional group concentration at a given time and *A*_*t*_ is the area of the absorbance band at a given time.

Secondary amine concentration [SA]_*t*_ is calculated using the previously determined primary amine concentration in eq 6
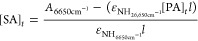
6

There is no suitable absorption peak in NIR associated with tertiary amines, as no flexible N–H bonds are present. Therefore, the tertiary amine concentration must be calculated using the primary and secondary amine concentrations shown in eq 7.
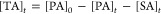
7where [TA]_t_ is the tertiary amine concentration at a given time.

Another calculation used by St John and George was to determine the excess epoxide reactions, that is, epoxide reactions not involving an amine (assumed to be etherification). The initial epoxide concentration, [EP]_0_, is reduced due to epoxide amine reactions leaving an excess of epoxide groups [EP^a^]_*t*_ free to participate in other reactions, as defined in eq 8.
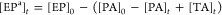
8

### 4.4 Network Formation

**Figure 5:**
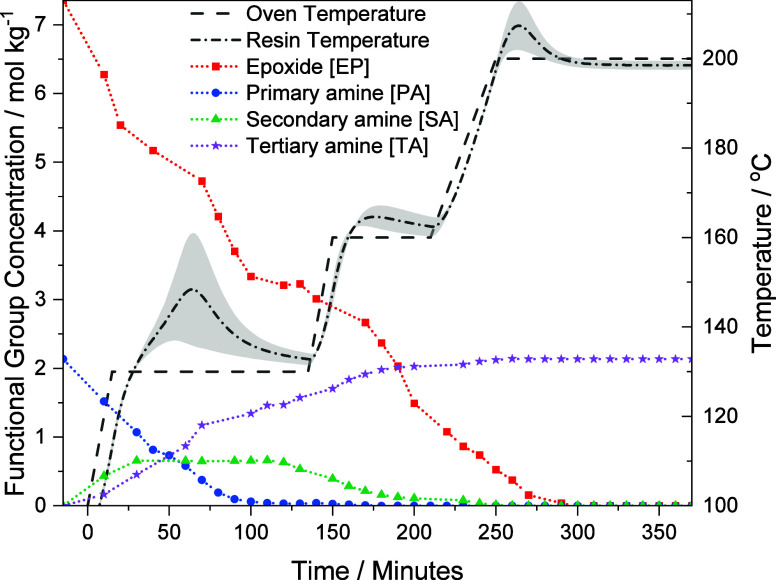
Functional group concentration (smoothed) and resin temperature profile of TG*m*AP/33′DDS. The resin temperature error band given is the standard deviation.

Cure monitoring of TG*m*AP/33′DDS via NIR and resin temperature is presented in Figure 5. The initial functional group concentrations, as determined above, are the starting points for the curing reaction. This is taken to be at −15 min, which reflects the approximate time taken to mix the reactants. The first NIR measurements are taken just after 0 min and will not have the same concentration values due to reactions occurring during the mixing stage. During the mixing stage and the first temperature ramp, there is a sharp decrease in [EP] and a decrease in [PA]. At 15 min, the 130 °C dwell is reached and the rate of EP and PA consumption slows down due to the reduced mobility of unreacted groups attached to the same molecule. Despite coinciding with a slower rate of EP consumption, a peak in the resin temperature profile occurs, as the initial quick rate of reaction will have released energy as heat due to the exothermic nature of the epoxide ring opening reaction. This heat will build up in the resin cumulatively alongside the increase in oven temperature and keep building. Only when the rate of reaction slows down will the temperature peak and fall toward the oven temperature.

At 70 min, the rate of EP and PA consumption increases due to the resin temperature overshoot. This is supported by the formation of tertiary amines, as shown by the TA line in Figure 5, indicating that epoxide secondary amine reactions are occurring. At 100 min, the EP consumption rate slows down, coinciding with the almost entire consumption of PA. Therefore, the main reactions that can occur at this point are secondary amine or nonamine reactions. Between 100 and 150 min, approximately 0.1 mol kg^–1^ PA is consumed and [SA] decreases by 0.3 mol kg^–1^. This small consumption of PA and SA is accompanied by a stabilization of resin temperature to 5 °C above oven temperature, indicating that reactions are occurring but not as often as at the beginning of the 130 °C dwell.

The [SA] curve in Figure 5 peaks at approximately 0.7 mol kg^–1^. The maximum [SA] is 2.1 mol kg^–1^, suggesting that upon formation of secondary amines, many are consumed straight away to form tertiary amines, as shown by the increase in [TA] in the initial stages of the 130 °C dwell. If this did not happen, [PA] would fall to 0.0 mol kg^–1^ while [SA] would rise to 2.1 mol kg^–1^.

**Figure 6:**
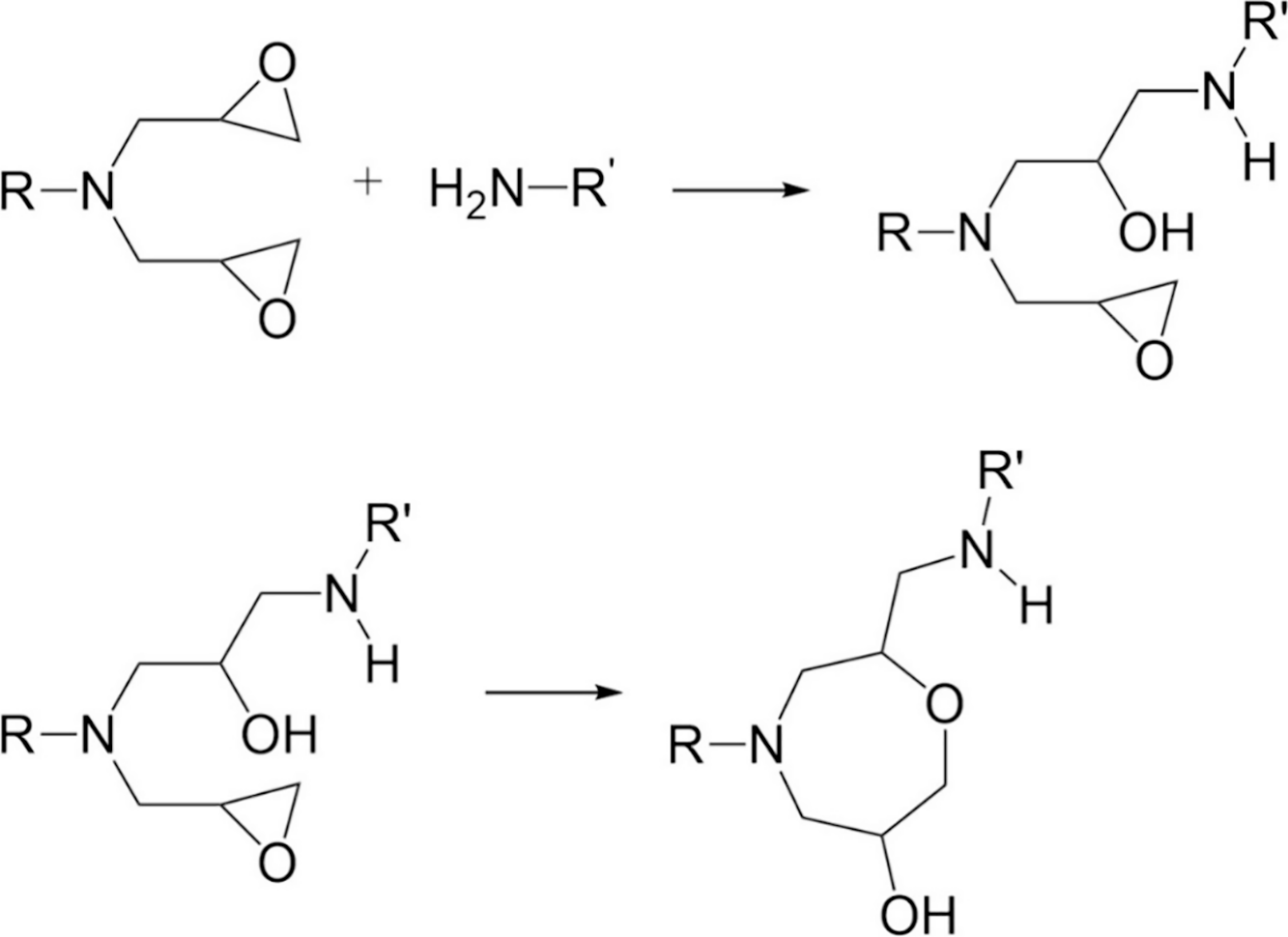
Internal cyclization etherification in TGAP.^40^

In the next ramp to 160 °C, there is substantial consumption of SA and EP, which coincides with a smaller resin temperature overshoot. By the end of the 160 °C dwell, a small amount of PA and SA remains. At 180 min, there is a significant drop in [EP], substantially more than would react with the PA and SA, indicating that another type of reaction is occurring. This can be assumed to be etherification as it is an epoxy-rich system, and substantial amounts of hydroxyl groups are available.^39^ However, the structure of TGAP has a glycidyl amine group on one side of the phenyl ring, placing two epoxide rings in close vicinity. If one reacts to give a hydroxyl group and the other does not, the possibility of an etherification reaction in the form of internal cyclization increases, as shown in Figure 6.^40,41^ Internal cyclization to form an ether link is a slow reaction and will only occur once all amine groups have reacted.^40^ There are no suitable peaks in NIR to identify if internal cyclization or etherification occurs as there is no net increase in hydroxyl groups.

The next ramp to 200 °C consumes any remaining SA, and as TA formation simultaneously stabilizes, a reduction in [EP] suggests that etherification occurs. This is paired with a small temperature overshoot caused by the cumulative buildup of energy from the remaining chemical reactions.

After this final resin temperature peak, [EP] stabilizes and there is very little concentration change in the final dwell, suggesting that very few reactions occur past this point. This can be confirmed *via* the resin temperature profile, which drops slightly below 200 °C, suggesting that no additional heat is being generated and the vitrified material is insulating the thermocouple.

The concentration values of EP, PA, and SA are 0 mol kg^–1^ at 370 min, suggesting that the degree of conversion is 100%, as no more possible reactants are left. However, this may be obscured by reduced signal-to-noise ratio due to overlap from hydrogen-bonded free OH in the secondary amine peak.^22^

**Figure 7:**
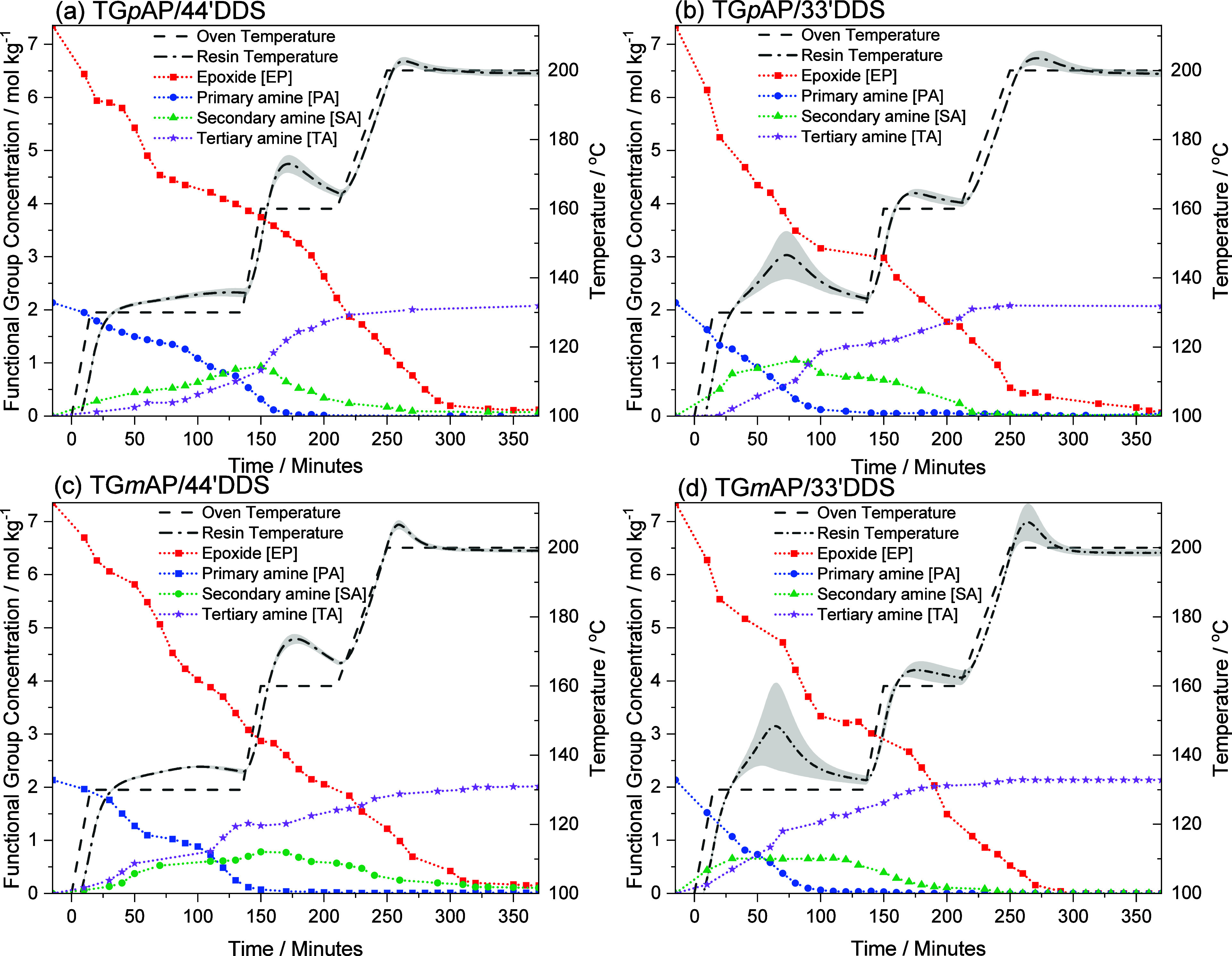
Functional group concentration (smoothed) and resin temperature profile of the four TGAP/DDS formulations as a function of time: (a) TG*p*AP/44′DDS, (b) TG*p*AP/33′DDS, (c) TG*m*AP/44′DDS, and (d) TG*m*AP/33′DDS. The error band given is the standard deviation.

The functional group concentrations and resin temperature profiles for all four TGAP/DDS formulations are listed in Figure 7. The *para–para*, *para–meta*, and *meta–para* formulations qualitatively display a similar series of events as described above for the *meta–meta* formulation.

### 4.6 Epoxide

**Figure 8:**
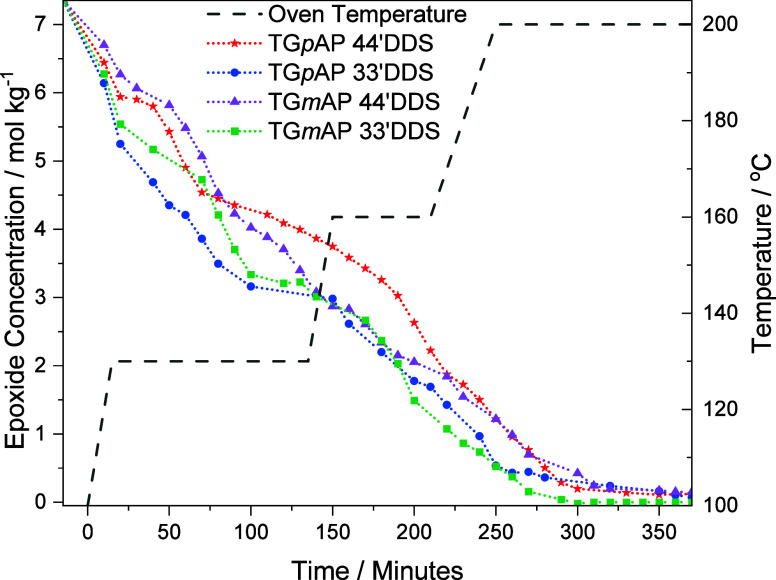
Epoxide concentration in the four TGAP/DDS formulations during cure (smoothed).

The comparison of epoxide consumption in the four different formulations is presented in Figure 8. On mixing at −15 min, EP consumption separates based on the hardener. Both TG*p*AP/33′DDS and TG*m*AP/33′DDS follow a similar EP consumption pattern in the first 30 min, and the same can be said for TG*p*AP/44′DDS and TG*m*AP/44′DDS.

**Figure 9:**
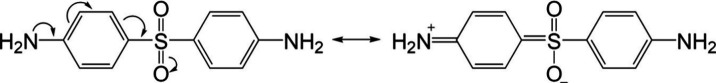
Lone-pair delocalization in 44′DDS.

The steeper gradient of [EP] change for 33′DDS formulations would suggest that 33’DDS is more reactive than 44′DDS. This is expected due to the electron-withdrawing effect of the sulfone group allowing for the delocalization of the nitrogen’s lone pair of electrons throughout 44′DDS, as shown in Figure 9. This is not possible in 33′DDS, and therefore the *meta* amine is a more effective nucleophile.

Considering this, EP consumption is quick upon mixing and during the first temperature ramp. Once the temperature dwell (130 °C) is reached at 15 min, EP consumption slows down in both the 33′DDS and 44′DDS formulations. At approximately 50 min, more EP has been consumed in the *para–para* formulation compared to the *meta–para* and similarly the *para–meta* compared to *meta–meta*. This is despite TG*p*AP being found to be generally more stable than TG*m*AP,^11^ suggesting etherification.

**Figure 10:**
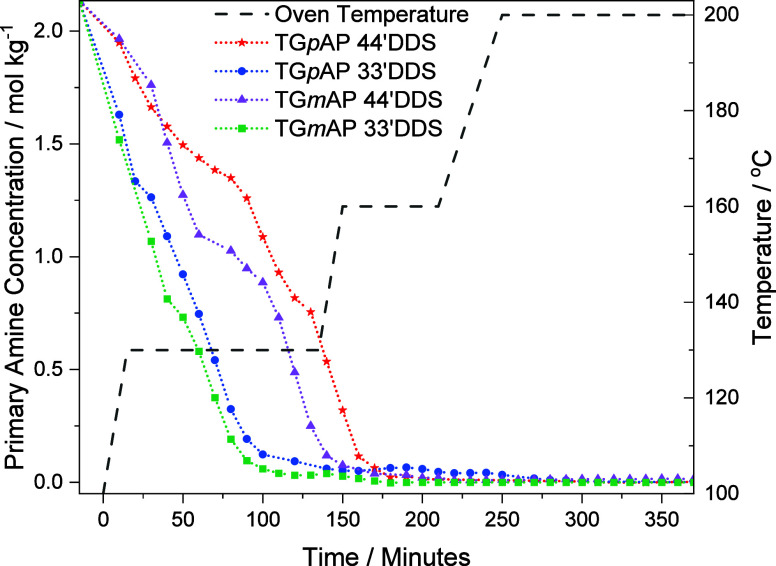
Primary amine concentration in the four TGAP/DDS formulations during cure (smoothed).

The two 33′DDS formulations continue to consume EP at a fast rate until 110 min, when the rate of EP consumption slows down. This reduction in consumption rate coincides with near total consumption of PA, as shown later in Figure 10. Returning to Figure 8, it can be seen that the 44′DDS formulations vary more during this time, both following a similar trend up to 80 min.

After approximately 150 min, TG*p*AP/33′DDS, TG*m*AP/44′DDS, and TG*m*AP/33′DDS [EP] follow the same trend until the end of the cure cycle. At 230 min, TG*p*AP/44′DDS follows the same trend as the other formulations until the end of the cure cycle.

Previously, the initial [EP] value for each formulation was determined to be 7.35 mol kg^–1^ and the final [EP] values are shown in Table 3. These results suggest that the 33′DDS formulations consume the greatest amount of epoxide, although the differences are relatively small. This is an epoxide-rich system; 2.14 mol kg^–1^ of primary amine was present at the start of the reaction, and therefore, 2.14 mol kg^–1^ of secondary amine was created during the cure schedule. Assuming every mole of primary and secondary amine was reacted, 3.07 mol kg^–1^ of epoxide is left in the system to react. From that, we may anticipate the final [EP] value to be related to the epoxy monomer rather than the type of hardener used. The data in Table 3 suggest that it is a combination of epoxide and hardener monomer that influences the final [EP]. The difference between the isomers of both epoxy and amine monomers is in the position of the reactive groups on the phenyl ring(s). The molecular shape of TG*p*AP and 44′DDS is more linear than in TG*m*AP and 33′DDS, which results in more conformational freedom in the latter.

**Table 3:** Final Epoxide Concentration Values for the Four TGAP/DDS Formulations

	TG*p*AP/44′DDS	TG*p*AP/33′DDS	TG*m*AP/44′DDS	TG*m*AP/33′DDS
final [EP]/mol kg^–1^	0.12	0.07	0.15	0.00

The presence of a *meta* epoxy, *meta* amine, or both therefore increases the degrees of freedom available and may explain why the EP consumption slows down in TG*p*AP/44′DDS. Varley et al. used the Flory equation to calculate the gelation point to be 41% in near-stoichiometric TG*p*AP/44′DDS,^18^ whereas an epoxy-rich formulation has been used here and 41% refers to overall conversion rather than epoxide conversion. This gelation point would correspond to approximately 4.3 mol kg^–1^ [EP], which is the point at which the TG*p*AP/44′DDS [EP] line starts to deviate from the other formulations. At the point of gelation, mobility is starting to be restricted, meaning rather than moving as free oligomers or monomers, they are starting to cross-link, and their movement is restricted to pivoting about a fixed point or similar short-range motions. A more linear system such as TG*p*AP/44′DDS will leave unfilled volume in the network as a result of cross-linking, and the distance between reactive groups will be significant. In contrast, a more nonlinear molecule such as TG*m*AP/33′DDS will be able to fill this volume to a greater extent and reactive groups will be in closer proximity.

### 4.7 Primary Amine

The comparison of primary amine consumption in the four different formulations is presented in Figure 10. The 33′DDS formulations behave similarly and are consumed significantly quicker than the 44′DDS formulations. TG*p*AP/44′DDS consumes more PA than TG*m*AP/44′DDS initially. At 40 min, the rate of PA consumption increases in TG*m*AP/44′DDS whereas in TG*p*AP/44′DDS, the rate decreases. The TG*m*AP formulations consume PA quicker than the TG*p*AP equivalents, suggesting that they are more reactive.

Each formulation has a point in its PA consumption where the rate of consumption slows. In the two 33′DDS formulations, the slowing of the PA consumption rate occurs much earlier than the 44′DDS formulations. In the 33′DDS formulations, significant epoxy primary amine reactions are possible at 130 °C, whereas in 44′DDS, it is less likely as the rate of PA consumption is slower.

Figure 10 shows, for the 44′DDS formulations, that TG*m*AP consumes PA quicker initially than TG*p*AP, most likely due to a slightly greater reactivity in TG*m*AP compared to TG*p*AP. In TGAP, the phenylene substituent position influences the reactivity of the epoxy groups to a lesser extent than similar effects observed in DDS. The ability to delocalize, which causes significant substituent effects in DDS, is not available in TGAP. Also, any electron-withdrawing effects caused by the TGAP amine or ether groups are not significant enough to substantially influence epoxy reactivity, all of which results in similar reactivity to PA in both *meta* and *para* TGAP. The rate of PA consumption for TG*m*AP slows down at approximately 70 min compared to TG*p*AP at about 40 min. The magnitude of each formulation’s resin temperature overshoot is different, with TG*m*AP being greater as more PA has been consumed. The resin temperature being higher than the oven temperature allows for an increase in the rate of PA consumption toward the end of the dwell. The increase in the rate of PA consumption for TG*p*AP/44’DDS occurs at 90 min compared to TG*m*AP/44′DDS, which occurs at approximately 110 min. This is expected as there is a greater proportion of PA in the TG*p*AP/44′DDS system and, therefore, a greater probability of reactions due to more reaction sites.

Upon reaching near complete consumption between 100 and 180 min for all formulations, [PA] stays relatively stable. TG*p*AP/33′DDS shows a slight increase at 180 min, but this effect is small and situated between a hydroxyl band at 4900 cm^–1^ and another at 5200 cm^–1^. The band at 4900 cm^–1^ has been reported to cause a baseline shift,^22^ which could introduce inaccuracy into the absorbance measurement for the band, resulting in an unexpected [PA] increase. An increase in [PA] is not possible in an epoxy amine curing reaction.

### 4.8 Secondary Amine

**Figure 11:**
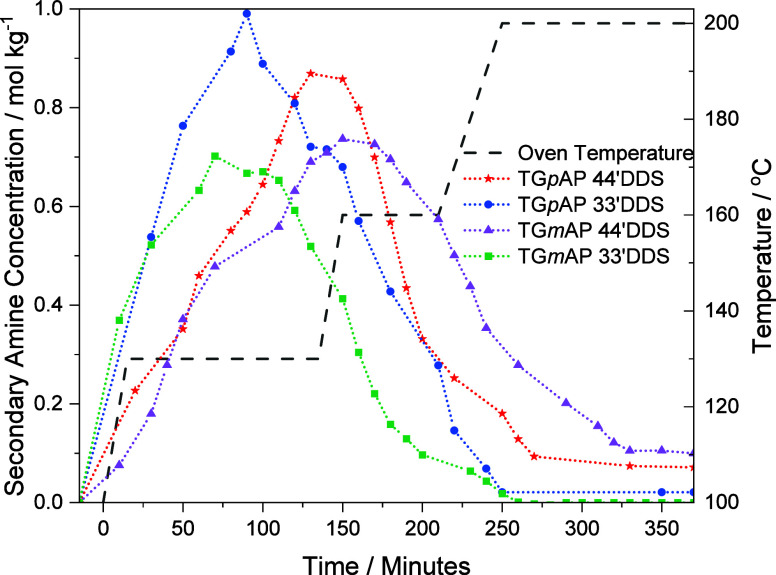
Secondary amine concentration in the four TGAP/DDS formulations during cure (smoothed).

Unlike epoxide and primary amines, secondary amines do not exist in the starting reagents but are produced during the reaction between the epoxide and primary amines. Following this reaction, secondary amines react with epoxides to produce tertiary amines. These two characteristics of secondary amines mean that monitoring [SA] is different from monitoring [PA] and [TA]. The [SA] at a particular time does not indicate how much SA has reacted to form TA, so a limited amount of information is available. The comparison of secondary amine concentration in the four different formulations is presented in Figure 11. The two 33′DDS formulations follow a similar trend to each other, as do the two 44′DDS formulations, a feature also observed in the primary amines. The following trend based on the hardener continues throughout the curing process, but there is a significant amount of crossover between *para* and *meta* epoxy formulations before the peak of each curve.

Each formulation’s [SA] peak is considerably lower than the maximum [SA] possible (2.14 mol kg^–1^). The formulation with the highest [SA] value is TG*p*AP/33’DDS, which peaks at approximately 1.0 mol kg^–1^. The peak [SA] value is significant, as it indicates the amount of TA present in the network (where [PA] is taken into account via eq 7). A [SA] peak of 1.0 mol kg^–1^ shows that the conditions of the first dwell (130 °C) were suitable for secondary amine epoxide reactions to occur. If there were no further reactions after the epoxide-primary amine reaction, then [SA] would be the inverse of [PA] and would reach a maximum of 2.14 mol kg^–1^. Other studies have found that [SA] does reach the maximum possible [SA] although generally, these have been in bifunctional glycidyl ether resin systems.^24^ In contrast, in this study, and in others such as Janisse and Wiggins,^42^ it is observed that [SA] does not reach the maximum value.

Table 4 shows the remaining concentration of secondary amine in each TGAP/DDS formulation where only TG*m*AP/33′DDS consumes all SA. A similar pattern exists here as was observed in the final epoxide concentration in Table 3. Similarly, the relative position of the reactive groups on the phenyl ring influences their mobility. The molecules with greater freedom (the nonlinear 33′DDS and TG*m*AP) have an increased probability of fully reacting.

**Table 4:** Final Secondary Amine Concentration Values for the Four TGAP/DDS Formulations

	TG*p*AP/44′DDS	TG*p*AP/33′DDS	TG*m*AP/44′DDS	TG*m*AP/33′DDS
final [SA]/mol kg^–1^	0.08	0.02	0.10	0.00

Comparing the resin temperature profiles for the four formulations in Figure 7 reveals several differences resulting from substituent effects. The 33′DDS formulations have a significant temperature overshoot during the first dwell compared to the 44′DDS formulations. In contrast, the 44′DDS formulations have a significant temperature overshoot in the second dwell. Figures 10 and 11 show that the larger 33′DDS overshoots primarily correspond to PA reactions whereas the larger 44′DDS overshoots primarily correspond to SA reactions. While the DDS substituent influence on temperature overshoots is significant, there is relatively little TGAP substituent influence observed, where the temperature overshoots seen in Figure 7 are similar for TG*p*AP compared to TG*m*AP. This relates to the differences in reactivity between the 44′ and 33′DDS discussed earlier, which contrast with the less substantial reactivity differences between TG*p*AP and TG*m*AP. Generally, the temperature overshoots increased the rate of reaction but there is no indication that they change the mechanism of cure.

### 4.9 Tertiary Amine

**Figure 12:**
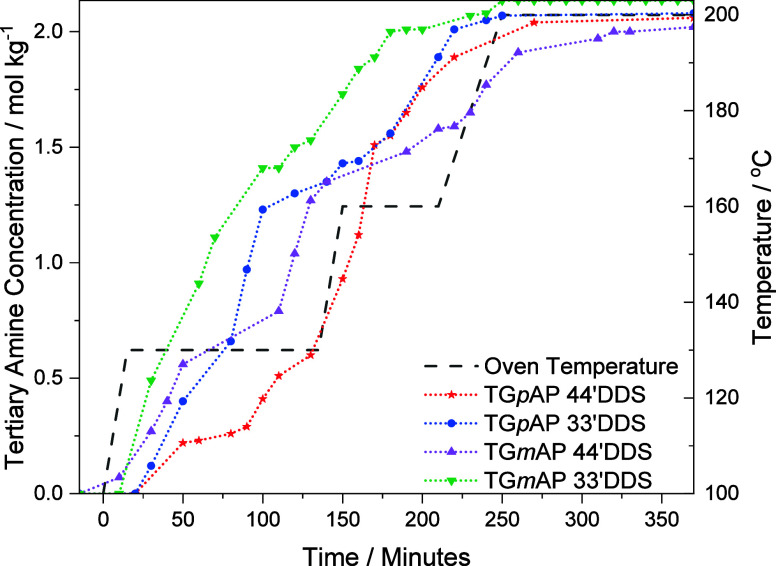
Tertiary amine concentration in the four TGAP/DDS formulations during cure (not smoothed).

The comparison of tertiary amine formation in the four different formulations is presented in Figure 12. Initially, TG*m*AP/33′DDS forms the most TA, TG*p*AP/33′DDS and TG*m*AP/44′DDS show a similar trend, forming similar amounts of TA to each other, and TG*p*AP/44′DDS forms less TA at the beginning until approximately 170 min. This indicates that in TG*m*AP/33′DDS, and to an extent in TG*p*AP/33′DDS and TG*m*AP/44′DDS, significant cross-linking occurs at the start of the reaction. This leads to localized areas of cross-linking^42^ due to the mobility and hence proximity of lower molecular weight species. As the curing reactions continue, these areas of higher cross-link density are then connected via areas of lower cross-link density, which would result in a less homogeneous cross-linked network. In TG*p*AP/44′DDS, tertiary amine formation occurs more slowly, creating a more homogeneous cross-linked network as more linear bonds are formed initially. Similar findings were found by Sahagun and Morgan when curing DGEBA/33′DDS at different temperatures.^43^

Table 5 shows the glass transition temperature for each formulation.^11^ It has previously been reported that in an inhomogeneous network, with a mixture of low and high cross-link density areas, a lower glass transition temperature (*T*_g_) is observed.^43,44^ Similarly, a homogeneous network has consistent areas of high cross-link density, resulting in a higher *T*_g_ value. TG*p*AP/44′DDS has the highest *T*_g_ of all the formulations, TG*m*AP/33′DDS has the lowest, and TG*m*AP/44′DDS and TG*p*AP/33′DDS have similar values. Also shown in Table 5 are the areas of the low temperature beta transition for each formulation.^11^ The area of the beta transition gives an indication of the free volume in the network. TG*p*AP/44′DDS has the highest beta transition area, suggesting it has the highest free volume space, which is expected as it is the most homogeneously cross-linked network but also the formulation with the most linear starting reagents.

**Table 5:** Glass Transition Temperatures (*T*_g_) and Beta Transition Areas for the Four TGAP/DDS Formulations Taken from Ramsdale-Capper and Foreman^11^^a^

	TG*p*AP/44′DDS	TG*p*AP/33′DDS	TG*m*AP/44′DDS	TG*m*AP/33′DDS
*T*_g_/°C	270 ± 1	231 ± 1	237 ± 1	212 ± 1
area of beta transition	3.43 ± 0.26	3.08 ± 0.33	2.86 ± 0.16	2.32 ± 0.22

a The error given is the standard deviation.

### 4.10 Other Epoxide Reactions

**Figure 13:**
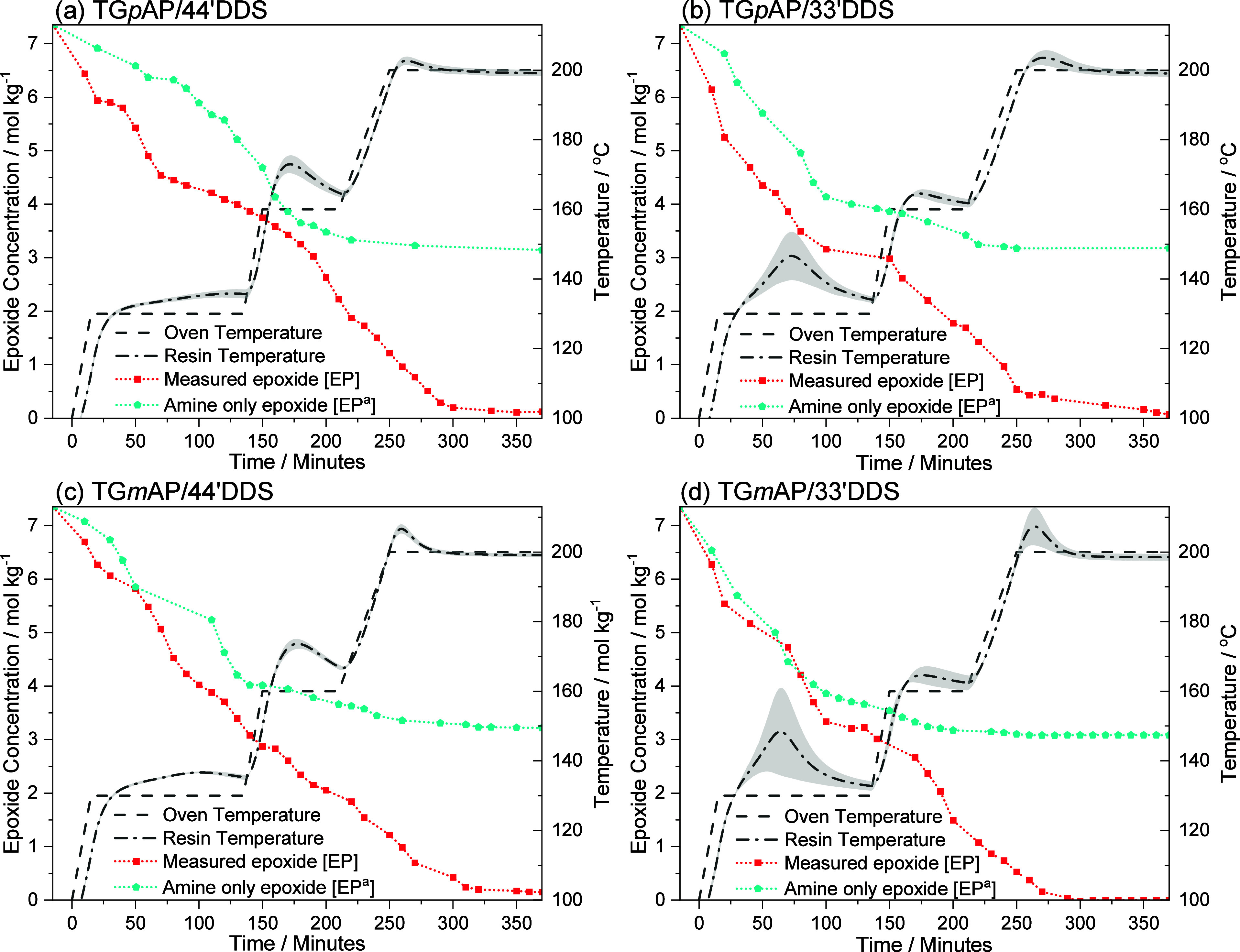
Nonamine [EP] vs measured [EP] (smoothed) and resin temperature profile of the four TGAP/DDS formulations as a function of time: (a) TG*p*AP/44′DDS, (b) TG*p*AP/33′DDS, (c) TG*m*AP/44′DDS, and (d) TG*m*AP/33′DDS. The error band given is the standard deviation.

Epoxy amine reactions are not the only reactions that occur during the epoxy amine network formation. While NIR analysis is unable to directly quantify these reactions, it can be used to identify whether EP is consumed without the consumption of PA and SA. In Figure 13, nonamine epoxy reactions are estimated by plotting the difference between [EP] and [EP^a^]. This technique is not exact, and there is evidence of inaccuracy in both (c) TG*m*AP/44’DDS and (d) TG*m*AP/33′DDS where the two lines cross over. This should be impossible and could be caused by deconvolution inconsistency.

Figure 13 initially shows the epoxy monomers following similar trends rather than the amines. Initially, a significant gap exists between [EP] and [EP^a^] in both TG*p*AP formulations, showing that epoxide nonamine reactions occur early in the network formation via etherification. In the TG*m*AP formulations, the gap between [EP] and [EP^a^] is initially small, suggesting that most of the reactions are between epoxide rings and amines (primary and secondary) toward the start of the network formation.

If hydroxyl groups are present alongside tertiary amines, etherification can be catalyzed and occur at low temperatures.^45,46^ St John and George observed this in TGDDM but found that the tertiary amine only accounted for 10% catalytic activity.^19^ TGDDM is approximately twice the size of both TG*p*AP and TG*m*AP, so there is a possibility that a steric effect influences the catalytic efficiency of the tertiary amine in the former. Rocks et al. investigated curing glycidyl amine containing resins with an anhydride hardener at ambient temperatures and found that they could undergo curing reactions. TG*p*AP was found to be less reactive than TGDDM, but this was because the former has one glycidyl amine group compared to the latter two.^45^ TG*m*AP was not investigated; therefore, its reactivity with anhydrides is unknown.

**Figure 14:**
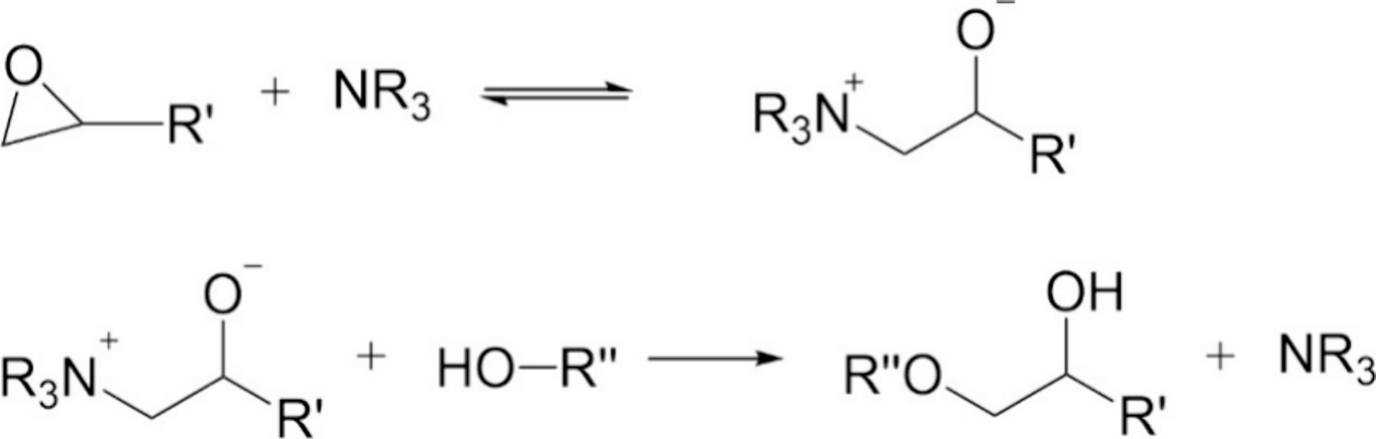
Reaction pathway of etherification catalysis via a tertiary amine.^47^

The difference between TG*p*AP and TG*m*AP is the position of the glycidyl amine on the phenyl ring. TG*p*AP formulations undergo more nonamine reactions initially, suggesting that the *para* position of the glycidyl amine group influences this. For those nonamine reactions to occur, the tertiary amine in the glycidyl amine of TGAP has to interact with a hydroxyl or another epoxide. Hydroxyl groups will be available, as some epoxide primary amine reactions occur initially, as shown in the NIR functional group analysis. The reaction pathway for tertiary amine catalysis of etherification is shown below in Figure 14.^47^

The findings in Figure 13 suggest that TG*p*AP’s glycidyl amine behaves as a more effective tertiary amine catalyst, promoting etherification reactions at the start of the curing reaction, whereas this is less likely in TG*m*AP. This affects how the network forms and can be shown by the *T*_g_ values. Varley et al. suggest a *para* ether will activate an amine more readily than a *meta* ether.^10^ If the glycidyl amine portion of the epoxy is involved in forming an ionic catalyst structure, it is less likely to be involved in any epoxy amine reactions in the first dwell. Therefore, the portion of TG*p*AP involved in forming bonds is the glycidyl ether (epoxy amine reactions still occur at this point). In TG*p*AP/44′DDS, the extent to which these nonepoxy reactions occur is initially higher than in TG*p*AP/33′DDS as the PA rate of reaction is slower than 33′DDS.

**Figure 15:**
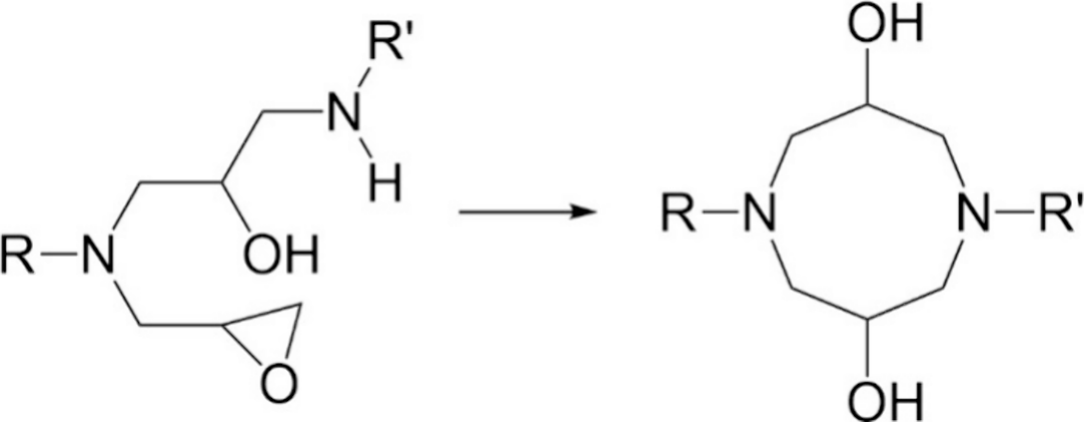
Internal cyclization via an epoxide and secondary amine to form an eight-membered ring.^40^

While the glycidyl amines may behave as tertiary amine catalysts in the TG*p*AP formulations, this is less likely to be the case in the TG*m*AP formulations, where the glycidyl amine portion is freer to react with the hardener. This opens up the possibility of internal cyclization during an epoxide secondary amine reaction, as shown in Figure 15.^40^ Once a primary amine reacts with a glycidyl amine epoxide ring, it forms a secondary amine. As there is another epoxide ring in close proximity, the secondary amine may react with it and form a cyclic structure. Internal cyclization would lead to a decrease in *T*_g_, as it forms a relatively flexible eight-membered ring instead of a cross-link. The evidence is shown in the *T*_g_ values in Table 5, where TG*m*AP formulations are lower than their TG*p*AP equivalents.

Noncatalyzed etherification occurs in all formulations when limited amines are available and there is a relatively high [EP]. It has been suggested previously that etherification occurs at high temperatures^48^ (approximately 200 °C), but Figure 13 shows that it starts to occur in the second dwell of the cure cycle at 160 °C. As the system is epoxy rich, etherification is possible at these low temperatures while also being catalyzed by the glycidyl amines in both TG*p*AP and TG*m*AP. This mechanism is not available in DGEBA, where etherification may not occur without a tertiary amine catalyst such as imidazole.^16^

## 5 Conclusions

The results from this study confirm that structural isomerism strongly influences the formation of TGAP/DDS networks, as previously suggested by Ramsdale-Capper and Foreman.^11^ This study found that two main contributing factors determined the formation of the network structure. The first factor is the structure of the hardener, with 33′DDS being the more reactive of the two isomers, consuming primary amine and secondary amine quickly as well as the significant resin to oven temperature difference shown in the temperature profile. Quick consumption of secondary amines led to areas of high and low cross-link density. The second factor is the structure of the epoxy monomer where TG*m*AP behaved differently to TG*p*AP. The evidence suggests TG*p*AP formulations underwent etherification reactions at lower temperature due to the increased catalytic behavior of the tertiary amine in *para* glycidyl amine. In contrast, TG*m*AP epoxy amine reactions on the glycidyl amine dominated at low temperatures, which probably formed some eight-membered rings due to internal cyclization.

This resulted in two different resin types, despite consisting of the same functional group components. The results suggest that TG*p*AP/44′DDS forms a more homogeneous network with less likelihood of internal cyclization while TG*m*AP/33′DDS forms a less homogeneous network. The nature of the analysis presented is necessarily influenced by the epoxy amine ratio and cure schedule. In a stoichiometric system, nonamine reactions may not readily occur in the earlier part of the reaction. The more industrially relevant epoxy-rich formulation used here suggests that etherification reactions occur at temperatures lower than those previously reported. Similarly, using a more industrially relevant cure schedule influences the types of reactions occurring. The transitions between multiple ramps and dwells during network formation are replicated in the functional group concentration profiles, allowing the differences between the isomeric formulations to be more clearly observed. Understanding the effects of these conditions allows us to fine-tune the network formation of resins and therefore tailor them to our desired properties.
